# Analysis of skin and corneal fiber electrodes for electroretinogram assessments in patients with major depressive disorder

**DOI:** 10.3389/fnins.2024.1501149

**Published:** 2024-11-25

**Authors:** Kathrin Nickel, Ludger Tebartz van Elst, Malina Beringer, Dominique Endres, Kimon Runge, Simon Maier, Sebastian Küchlin, Michael Bach, Katharina Domschke, Sven P. Heinrich, Evelyn B. N. Friedel

**Affiliations:** ^1^Department of Psychiatry and Psychotherapy, Medical Center - University of Freiburg, Faculty of Medicine, University of Freiburg, Freiburg, Germany; ^2^Eye Center, Medical Center - University of Freiburg, Faculty of Medicine, University of Freiburg, Freiburg, Germany; ^3^German Center for Mental Health (DZPG), Partner Site Berlin, Berlin, Germany; ^4^Faculty of Biology, University of Freiburg, Freiburg, Germany

**Keywords:** sensor strip skin electrodes, skin electrodes, fiber electrodes, corneal electrodes, RETeval^®^, electroretinogram, ERG, major depressive disorder

## Abstract

**Background:**

Electroretinograms (ERG) are usually recorded with non-invasive corneal electrodes, requiring direct contact with the ocular surface. However, corneal electrode application is not tolerated by some individuals. The advent of handheld ERG devices has facilitated the use of skin electrodes for ERG measurements. Skin electrodes do not require corneal contact and thus enhance patient comfort, simplify the attachment process, and reduce preparation time, which is particularly beneficial for clinical psychiatric research. Nevertheless, due to the different attachment methods, ERG amplitudes recorded with skin compared to corneal electrodes are considerably smaller. However, comparative data on ERGs recorded with skin vs. corneal electrodes in psychiatric populations are currently lacking.

**Materials and methods:**

We recorded flash electroretinograms of 57 healthy controls (HC) and 30 patients with a major depressive disorder (MDD) using both sensor strip skin and corneal electrodes with the handheld RETeval^®^ device.

**Results:**

The significant reduction in both the amplitude and peak time of the a-wave in MDD when using sensor strip skin electrodes could not be replicated with corneal electrodes. Comparing both electrode types in HC revealed a fair correlation between sensor strip and corneal electrodes for a- and b-wave amplitudes and a moderate correlation for a- and b-wave peak times.

**Conclusion:**

In addition to being better tolerated, sensor strip skin electrodes appear to be more effective than corneal electrodes in detecting ERG alterations in patients with MDD when using the RETeval^®^ device, making them a promising alternative to traditional corneal electrodes.

## 1 Introduction

The electroretinogram (ERG) is an established ophthalmological clinical examination method that assesses retinal function by recording the electrical activity of different retinal cells in response to a stimulation with flashes of light (fERG) (Robson et al., 2022). For fERG recordings, corneal fiber electrodes such as DTL (Dawson, Trick, and Litzkow) electrodes (Dawson et al., 1979) are frequently recommended. These are fine conductive silver-coated nylon threads that can be placed either along the lower eyelid in contact with the cornea or in the conjunctival sac.

The application of such fiber electrodes demands great attention, and patients have to be monitored carefully to ensure consistent positioning throughout the recording process (Hébert et al., 1999). Nevertheless, fiber electrodes are frequently used in clinical research because they record detailed retinal responses and demonstrate high stability (Kuze and Uji, 2000) and reproducibility (Hébert et al., 1999) in ERG measurements.

The manufacturer of the RETeval^®^ ERG system (LKC Technologies, Inc.) recommends the application of self-adhesive sensor strip skin electrodes rather than corneal electrodes for signal acquisition (LKC Technologies, Inc., 2023). The RETeval^®^ skin electrodes encompass ground, reference, and active silver-silver chloride electrodes in a single strip (Hobby et al., 2018; Man et al., 2020). Skin electrodes can easily be applied under the lower eyelid, which is more comfortable compared to corneal electrodes and substantially reduces preparation time. They are therefore particularly convenient for use in non-ophthalmological clinical routine examinations (Nakamura et al., 2016; Hobby et al., 2018), including measurements in patients with psychiatric disorders (Friedel et al., 2022a, 2022b).

Previous comparative studies found smaller amplitudes in skin vs. fiber electrode recorded responses (Mortlock et al., 2010; Tang et al., 2018), which is a potential drawback. However, variability seems to be only marginally higher (Mortlock et al., 2010) and recordings are less influenced by blinks (Tang et al., 2018).

In a previous investigation involving patients with manifest or suspected glaucoma and controls, the efficacy of fiber and sensor strip electrodes in recording the photopic negative response (PhNR) with the RETeval^®^ system was explored. The PhNR amplitude recorded via sensor strip electrodes exhibited a lower magnitude with a diminished signal-to-noise ratio (SNR) and a tendency toward an increased PhNR/b-wave ratio when compared to fiber electrodes. Nevertheless, both electrode types demonstrated comparable inter-session repeatability. It was concluded that while sensor strip electrodes may serve adequately for routine clinical assessments, fiber electrodes should be prioritized in cases of attenuated PhNR or elevated noise levels (Tang et al., 2018).

We previously reported results of fERG recordings in 30 major depressive disorder (MDD) patients (Friedel et al., 2024) in response to a PhNR stimulation (Frishman et al., 2018) with red flashes on blue background light using the RETeval^®^ system and sensor strip electrodes. A reduction in the a-wave amplitude was observed in patients with MDD compared to healthy controls (HC), alongside a trend-level reduction in the a-wave peak time (Friedel et al., 2024). In the same session, the participants of this study underwent a secondary fERG evaluation using a white flash on white background light for stimulation as recommended by the International Society for Clinical Electrophysiology of Vision (ISCEV) (Robson et al., 2022) and skin as well as fiber electrodes for recording.

In contrast to an ophthalmological setting, clinical research in psychiatric patients is particularly dependent on the ease and tolerability of the tests conducted, and personnel may have less experience in the application of corneal electrodes. Comparative data on the merits of fiber vs. skin electrodes in the setting of psychiatric patients are therefore needed.

The aim of this study was to evaluate whether sensor strip skin electrodes and fiber electrodes are equally suitable for detecting a-wave amplitude reductions in the fERG of patients with MDD using the RETeval^®^ device (LKC Technologies, Inc.) or if fiber electrodes should be prioritized due to their superior sensitivity.

We examined 57 HCs and 30 patients with an MDD, using both types of electrodes with the RETeval^®^ device. We sought to determine whether the previously identified reduction in the a-wave amplitude in these patients, observed with sensor strip electrodes and a red flash stimulation (Friedel et al., 2024), would be similarly or more pronounced using fiber electrodes and/or a white flash stimulation, as recommended by the ISCEV (Robson et al., 2022).

## 2 Materials and methods

### 2.1 Participants

The study followed the principles of the Declaration of Helsinki, with written informed consent from all participants. Ethics approval was obtained from the Ethics Committee of the University of Freiburg (Approval ID: 314/18). Initially, 67 HCs without any psychiatric disorder and 41 patients diagnosed with MDD according to the criteria of the Diagnostic and Statistical Manual of Mental Disorders, Fifth Edition (DSM-5) or the International Classification of Diseases, 10th revision (ICD-10) were recruited. This included individuals with a severe depressive episode (ICD-10: F32.2) or a recurrent depressive disorder, current episode severe (ICD-10: F33.2). Patients with MDD were diagnosed by an experienced senior psychiatrist from the outpatient clinic of the Department of Psychiatry and Psychotherapy of the University Medical Center Freiburg. Demographic and psychometric data of HCs and patients with MDD eligible for inclusion in the final analysis are summarized in Table 1. fERG examinations of these MDD patients using a PhNR stimulation (red flash) and the reported reductions in a-wave amplitudes in MDD have been published elsewhere (Friedel et al., 2024).

**Table 1:** Demographic and psychometric data from HC and MDD are summarized as median (first and third quartiles), range, number of participants (N), and proportions (in %).

Parameter	HC (*N* = 57)	MDD (*N* = 30)	*p*-value
Sex: female/male	43 (75%)/14 (25%)	21 (70%)/9 (30%)	0.6
Age in years	31 (25, 40); 19–65	32 (22, 39); 19–65	0.4
ICD-10 Diagnosis: F32.2/F33.2		16 (53%)/14 (47%)	
MADRS		36 (35, 40); 28–45^≠≠^	
BDI	2 (0, 4); 0–13	27 (22, 39); 14–53^≠≠^	<0.001
AQ	22 (18, 24); 9–27	20 (16, 22); 9–28^≠≠^	0.046
EQ	46 (44, 48); 30–59^≠≠^	44 (38, 49); 27–53^≠≠^	0.3
WURS-k	8 (4, 14); 0–34^≠^	11 (4, 26); 0–53^≠≠^	0.3
Antidepressant medication: yes/no		21 (70%)/9 (30%)	
SSRI		2 (10%)	
SNRI		4 (19%)	
Mirtazapine		6 (29%)	
SSRI+Mirtazapine		4 (19%)	
SNRI+Mirtazapine		5 (24%)	
Days of medication		7 (4, 11); 1–14	

Wilcoxon and Fisher’s exact tests were used for comparisons. AQ, Autism Spectrum Quotient (Baron-Cohen, 2001); BDI-II, Beck Depression Inventory-II (Beck, 1996; Hautzinger, 2006) EQ, Empathy Quotient (Baron-Cohen, 2004); F32.2, severe depressive episode without psychotic symptoms (ICD-10); F33.2, recurrent depressive disorder, current episode severe without psychotic symptoms (ICD-10); HC, healthy controls; ICD-10, International Statistical Classification of Diseases and Related Health Problems version 10; MADRS, Montgomery-Åsberg Depression Rating Scale (Montgomery, 1979); MDD, patients with major depressive disorder; N, number of participants; WURS-k, Wender Utah Rating Scale (Retz-Junginger et al.,2002); ≠, 1 or ≠ ≠, 2 missing data sets.

The following assessments were conducted for HCs and patients with MDD: the Beck Depression Inventory (BDI-II) (Beck et al., 1996; Hautzinger et al., 2006), the Autism Spectrum Quotient (AQ) (Baron-Cohen et al., 2001), the Empathy Quotient (EQ) (Baron-Cohen and Wheelwright, 2004), the Wender Utah Rating Scale (WURS-k) (Retz-Junginger et al., 2002), the Structured Clinical Interview for DSM (SCID-I and –II) (Wittchen et al., 1997), and the Symptom Checklist (SCL-90-R) (Derogatis and Savitz, 1999). For MDD patients, the Montgomery-Åsberg Depression Rating Scale (MADRS) (Montgomery and Åsberg, 1979) was additionally administered by an experienced senior psychiatrist to assess the severity of depressive symptoms.

The following exclusion criteria were defined for all study participants: age less than 18 or above 65 years, the presence of somatic conditions (such as diabetes mellitus, arterial hypertension, and seizures), or ophthalmological (apart from correctable refractive errors) diseases. We also excluded participants with a best corrected monocular decimal visual acuity worse than 0.8, assessed using the Freiburg Visual Acuity and Contrast Test (FrACT) (Bach, 2007), hyperopia exceeding +6D, myopia exceeding −6D, or incidental findings on optical coherence tomography (OCT) in either eye that required further clarification from an ophthalmologist.

Moreover, HCs with BDI-II scores >13 were also excluded. Additionally, MDD patients taking antidepressant medication other than classes of serotonin–norepinephrine reuptake inhibitors (SNRI) such as venlafaxine and selective serotonin reuptake inhibitors (SSRI) such as sertraline or mirtazapine were further excluded from the study.

### 2.2 fERG stimulation and recording

The handheld RETeval^®^ device (LKC Technologies, Inc., Gaithersburg, USA, firmware version 2.13.1) was used for stimulation and recording of light-adapted [10 min under normal room lighting conditions (500 lux)] fERG. The RETeval^®^ device enables Troland(Td)-based stimulation protocols where the device’s internal camera measures pupil size during recording and automatically adjusts flash intensities accordingly, eliminating the need for mydriasis.

Following the recommendations of the ISCEV (Robson et al., 2022), 100 white flashes with a flash strength of 85 Td·s (⩯3 cd·s/m^2^) were presented on 848 Td (⩯30 cd/m^2^) background light with a stimulation frequency of 2 Hz. A red LED in the RETeval^®^ device served as a fixation target during the recording.

All participants completed two recording sessions, one with DTL-style (Dawson et al., 1979) fiber electrodes (Manufacturer Spes Medica Italy, supplied by GVB geliMED GmbH Germany) and one with sensor strip electrodes supplied by the manufacturer of the RETeval^®^. The order in which electrodes were used was balanced between participants in both groups. The measurements were carried out consecutively, with only a few minutes apart. Before electrode placement, participants’ skin was cleaned using Nuprep^®^ skin preparation gel. Fiber electrodes were placed at the lower eyelids of both eyes with contact to the cornea as described by Bach (2024). Gold-cup reference electrodes were positioned at the ipsilateral canthi using conductive gel for impedance reduction. An ear clip, attached together with a moistened swab to one earlobe, served as a ground electrode.

The self-adhesive sensor strip skin electrodes were applied following the manufacturer’s instructions. After skin preparation with Nuprep^®^ skin preparation gel, the sensor strip electrodes were placed 2 mm under the lower eyelids of both eyes, aligning the nasal ends of the electrode strips horizontally with the center of the pupils.

The device’s internal signal processing encompasses a zero-phase 0.3 Hz high-pass filter to reduce electrode drift and offset while preserving waveform timing. Signals from multiple flashes are then combined using a trimmed mean to enhance the SNR, with outlier replicates (amplitudes exceeding 1 mV) removed prior to averaging. The resulting waveform undergoes wavelet-based denoising as described by Ahmadi and Rodrigo (2013), where wavelet components are selectively attenuated based on the SNR power ratio between the post-stimulus (signal) and pre-stimulus (noise) waveform segments (LKC Technologies, Inc., 2023).

### 2.3 Data preparation and statistical analysis

The quality of all fERG recordings was individually visually checked for proper peak detection, baseline drifts, or artifacts. Individual fERG recordings of single eyes were excluded prior to data analysis in case of artifact contaminations (excessive blinking, baseline drifts, etc.), incidental OCT findings, or uncorrectable low visual acuity. The RFF Extractor^®^ software (version 2.12.0.0) was used to extract a- and b-wave peak amplitudes in μV and corresponding peak times in ms. The a-wave amplitude was defined as the minimum voltage in relation to the baseline, and the b-wave amplitude as the voltage from the a-wave minimum to the b-wave maximum voltage. Additionally, the number of rejected flashes and the SNR were extracted. The SNR is internally defined by the device as the b-wave amplitude divided by the standard deviation (SD) of the pre-stimulus baseline after a linear fit subtraction has been applied to eliminate drifts.

Subsequent data preparation, statistical analysis, and graphical representations were conducted with “R” in Rstudio (Posit Team, 2023), using the “tidyverse” (Wickham et al., 2019) core packages.

We further computed the b/a amplitude ratio as a supplementary fERG parameter.

For participants contributing recordings from both eyes, data were averaged. Electrode data for both groups were summarized using the median, the arithmetic mean and SD, and corresponding 95%-confidence intervals. The magnitudes of differences between the compared data sets were calculated as proportional deviations in % for medians and means, respectively.

Based on previous findings (Friedel et al., 2024), we defined the a-wave amplitude and peak time as primary outcome variables and all other fERG parameters as secondary outcome variables. Hypothesis testing and effect size estimations were conducted using non-parametric Wilcoxon tests and, for comparison, parametric *t*-tests, along with Cohen’s *d* for effect sizes. Deviations from normality, assessed by Shapiro–Wilk tests, were noted accordingly. The significance level was defined as *α* = 0.05 and adjusted using a false discovery rate (FDR) procedure (Benjamini and Hochberg, 1995) for the primary and secondary outcome variables separately.

#### 2.3.1 fERG parameters in patients with MDD compared to HCs

Unpaired (two-sample) Wilcoxon and *t*-tests were used to compare primary and secondary fERG parameters between patients with MDD and HCs. Based on our previous findings (Friedel et al., 2024), we assumed the amplitude of the a-wave to be attenuated in MDD and conducted one-sided tests for this parameter. All other comparisons were based on two-sided tests without any assumptions. Effect sizes for group differences were estimated using Wilcoxon’s *r* and Cohen’s *d*, respectively.

#### 2.3.2 Sensor strip vs. fiber electrodes in HCs

In HCs, paired (one-sample) Wilcoxon and *t*-tests were calculated to compare primary and secondary fERG parameters recorded with both types of electrodes. Effect sizes were computed using Wilcoxon’s *r* and Cohen’s *d*, respectively (Tomczak and Tomczak, 2014). Based on previous reports (Mortlock et al., 2010; Tang et al., 2018), we assumed higher a- and b-wave amplitudes for recordings with fiber compared to skin electrodes and conducted one-sided tests for those parameters. Two-sided tests were performed for all other fERG parameters. Spearman’s correlation coefficients (*rho*) were further computed to analyze data from fiber and sensor strip electrodes in HCs.

## 3 Results

### 3.1 Participants

Table 1 summarizes the demographic and psychometric data of all study participants included in the final analysis. Of the 67 initially recruited HCs, 10 were excluded: 2 refused participation, 1 showed elevated BDI-II scores (>13), and 7 had incidental OCT findings in both eyes.

In the remaining 57 HCs, 14 individual eyes were excluded from analysis or were not available: One showed an OCT finding in one eye, three did not tolerate fiber electrode recordings at all, six fiber electrode recordings from single eyes had to be excluded due to poor recording quality (baseline drifts and excessive artifacts), preventing proper peak detection, and one sensor strip recording was excluded for the same reason.

Finally, 112 sensor strip and 101 fiber electrode eye recordings of 57 HCs were available, of whom 54 HCs provided measures with both electrodes (101 fiber electrode and 106 sensor strip eye recordings).

Of the initially recruited 41 MDD patients, 11 were excluded: three due to their antidepressant medication (bupropion, opipramol, and trimipramine), one because of uncorrectable low visual acuity in both eyes, one due to incidental OCT findings in both eyes, and six refused participation.

Of the remaining 30 patients with MDD, one had low visual acuity in one eye, another an OCT finding in one eye, and insufficient sensor strip recording quality in the other eye. Because of insufficient recording quality, additionally, one sensor strip and three fiber electrode eye recordings were also excluded from further analysis. Finally, two patients did not tolerate fiber electrode measurements at all.

Finally, of the MDD patients, 56 eyes recorded with sensor strip electrodes (N_MDD_ = 29) and 51 eyes recorded with fiber electrodes (N_MDD_ = 28) were included.

### 3.2 fERG parameters in MDD compared to HCs

**Figure 1:**
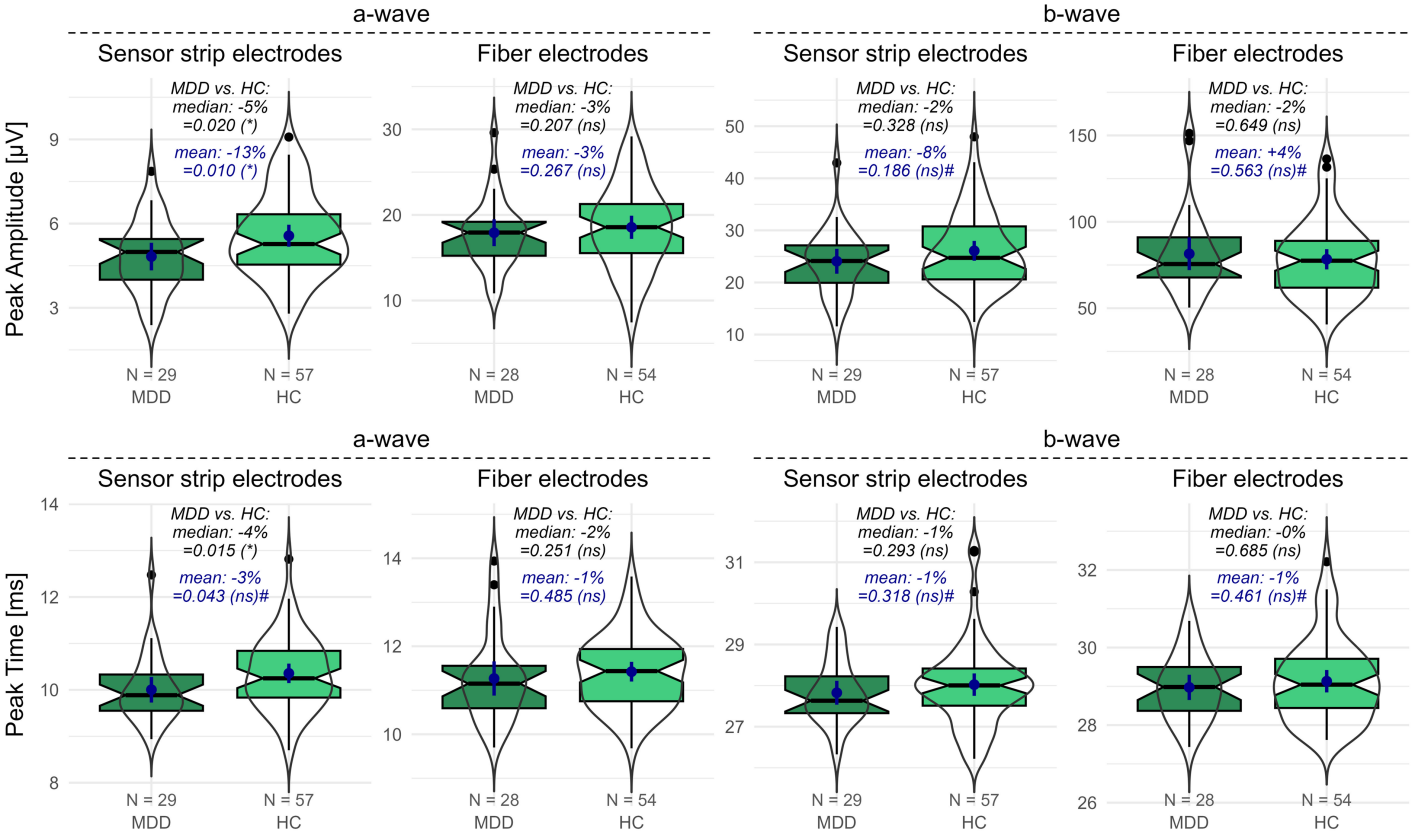
fERG parameters recorded with sensor strip and fiber electrodes in patients with MDD compared to HCs. Peak amplitudes (upper row) and peak times (lower row) of the a- and b-wave for patients with MDD and HCs were recorded using sensor strip and fiber electrodes. Boxplots indicate medians (black horizontal line within the box) and interquartile range (first and third quartiles; upper and lower ends of the boxes). Additionally, data distributions as violins and the means (blue dot) with the corresponding 95%-confidence intervals (blue vertical line) are depicted. *p*-values from the unpaired Wilcoxon and *t*-tests comparing patients with MDD and HCs are shown (# indicates non-normal distribution in at least one data set). Significance levels in brackets were FDR adjusted for the primary and secondary outcome variables separately. The proportional deviation of MDD medians and means from the HC medians and means in % (MDD vs. HC) is annotated. FDR, false discovery rate; HC, healthy controls; N, number of participants; ns, non-significant; MDD, patients with major depression; *, statistically significant; #, departures from normal distribution in at least one data set.

When measured with sensor strip skin electrodes, patients with MDD showed a reduced a-wave amplitude compared to HCs (one-sided Wilcoxon: *p* = 0.020; one-sided *t*-test: *p* = 0.010). This effect could not be replicated with fiber electrodes. Considering the proportional deviations of the MDD compared to the HC data, the difference in means (patients with MDD vs. HCs: −13%) is more pronounced than the difference in medians (patients with MDD vs. HCs: −5%) (Figure 1).

The a-wave peak time did not differ between patients with MDD and HCs when applying fiber electrodes. In contrast, when applying sensor strip skin electrodes, a significantly reduced a-wave peak time was observed in patients with MDD compared to HCs in the non-parametric Wilcoxon test (*p* = 0.015; patients with MDD vs. HCs: −4%). However, the difference in the mean a-wave peak time from the parametric *t*-test did not survive FDR adjustment (*p* = 0.043). It must be considered that departures from normality were detected in the peak time data (Supplementary Table S1).

No differences were detected for the b-wave amplitude (Figure 1) or peak time between patients with MDD and HCs using either sensor strip or fiber electrodes (Supplementary Table S1).

Similarly, neither the b/a amplitude ratio nor the SNR differed between patients with MDD and HCs applying sensor strips or fiber electrodes. The number of rejected flashes was elevated in the sensor strip recordings in patients with MDD compared to HCs but had to be considered non-significant after FDR adjustment (Supplementary Table S1).

### 3.3 Sensor strip vs. fiber electrodes in HCs

In the 54 HCs for whom complete data sets of both electrodes were available, a- and b-wave amplitudes were significantly smaller (*p* < 0.001), the b/a amplitude ratio larger (*p* < 0.001), and peak times shorter (*p* < 0.001) when recorded with sensor strip compared to fiber electrodes (Supplementary Table S2).

The SNR was higher when recording with fiber in comparison to sensor strip electrodes (*p* < 0.001). The number of rejected flashes was lower for sensor strip compared to fiber electrodes (*p* < 0.001) (Supplementary Table S2).

**Figure 2:**
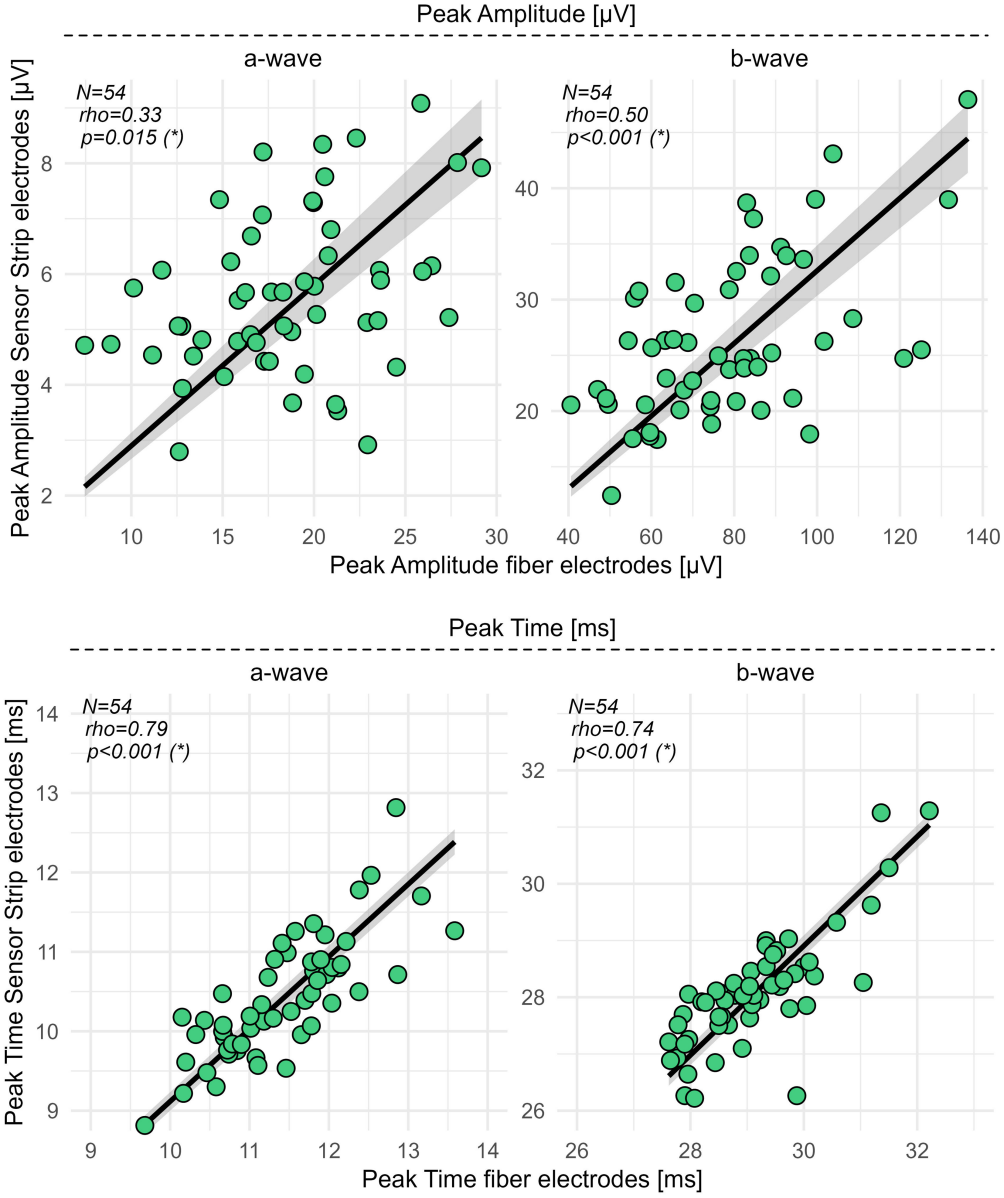
Correlation analysis for sensor strip vs. fiber electrodes in HCs. Peak amplitudes and peak times of the a- and b-wave from 54 HCs were recorded with sensor strip and fiber electrodes. Spearman’s *rho*, computed as the correlation coefficient, revealed fair correlations (0.3–0.5) (Chan, 2003) for the peak amplitudes of the a- and b-wave and moderate correlations (0.6–0.8) (Chan, 2003) for the a- and b-wave peak times. Significance levels in brackets were adjusted according to an FDR procedure considering primary and secondary outcome variables separately. FDR, false discovery rate; N, number of healthy controls; *rho*, Spearman’s correlation coefficient; *, statistically significant.

Spearman’s correlation coefficient (*rho*) revealed for the peak time of the a- (*p* < 0.001, *rho* = 0.79) and b-wave (*p* < 0.001, *rho* = 0.74) a moderate (Chan, 2003) correlation between fiber and sensor strip skin electrodes. For the amplitudes of the a- (*p* = 0.015, *rho* = 0.33) and b-waves (*p* < 0.001, *rho* = 0.50), fair correlations between both types of electrodes were detected (Figure 2; Supplementary Table S2).

## 4 Discussion

In patients with psychiatric disorders, sensor strip electrodes, which are easier to tolerate and simpler to use than corneal electrodes, could be of great advantage. However, ERG amplitudes recorded with skin electrodes are generally smaller, making quantifications of alterations more challenging (Robson et al., 2022). We report comparative data on sensor strip skin vs. fiber electrodes, including a first comparison in a psychiatric population.

In MDD patients, our previous study reported a significant reduction of the a-wave amplitude and a trend-level reduction in a-wave peak time when recording the fERG with the RETeval^®^ device using sensor strip skin electrodes and a red flash for optimal PhNR stimulation (Friedel et al., 2024).

In the current study, we aimed to determine whether group differences between MDD and HC are more pronounced using fiber electrodes and a standard white ISCEV flash (Robson et al., 2022).

Consistent with our previous observations using a red flash stimulation, there was an attenuation of the a-wave amplitude in MDD patients compared to HCs when recording the fERG in response to a white ISCEV flash with sensor strip electrodes. However, this finding was not replicated with fiber electrodes. Although the effect sizes were small, these differences were detectable with skin electrodes, which usually produce smaller amplitudes than corneal fiber electrodes (Robson et al., 2022).

Additionally, the reduced peak times of the a-wave in MDD patients were only observed with sensor strip and not fiber electrodes. Compared to the red flash stimulation, which showed trend-level reductions in a-wave peak times in MDD, the white ISCEV flash produced a significant group difference between MDD patients and HCs, suggesting its greater suitability for evaluating fERG peak times in MDD patients.

Our results of the electrode comparisons in HCs are in line with previous literature, reporting attenuated amplitudes (by approximately two-thirds) in the a- and b-wave of the fERG for skin electrodes in comparison to fiber electrodes (Coupland and Janaky, 1989; Esakowitz et al., 1993; Mortlock et al., 2010; Tang et al., 2018). However, only Tang et al. (2018) also used the RETeval^®^ device for fERG recordings and, similar to our observations, found higher SNRs [for the photopic negative response (PhNR)] with fiber compared to skin electrodes.

In accordance with previous studies, we observed shorter peak times for the a- (approx. –10%) and b-wave (−3%) with skin electrodes (Coupland and Janaky, 1989; Tang et al., 2018).

The number of flashes rejected during recording was higher for fiber than for the sensor strip electrodes, probably due to higher discomfort, which led to increased susceptibility to artifacts from blinking.

Spearman’s coefficients indicated a fair (*rho* 0.3–0.5) correlation between skin and fiber electrodes for the a- and b-wave peak amplitudes, while moderate correlations (*rho* 0.5–0.8) were observed for the b/a amplitude ratio and the a- and b-wave peak times. While correlation coefficients are widely used in medical research, they should always be interpreted with caution due to their susceptibility to range dependence (Holopigian and Bach, 2010).

Regarding the amplitudes, the fair to moderate congruence observed between recordings with sensor strip and fiber electrodes may be due to either the interaction between electrode location and the surface distribution of the electrical potentials and/or differences in the device’s internal signal processing when using electrodes with different SNRs for RETeval^®^ recordings. The very consistent 1-ms difference in average peak time is presumably also a device characteristic. After correcting for this, a spot check comparing the mean absolute peak time difference between electrodes to the mean absolute interocular differences (the latter as a proxy for test–retest variability) in binocularly tested participants showed that both variabilities are in the order of half a millisecond. The individual inter-electrode differences in peak time thus seem to not systematically exceed the expected test–retest variability. Moreover, we cannot exclude a confounding effect due to a different impedance between skin and corneal electrodes. This should be addressed in future studies.

We assume that the significant group differences between MDD patients and HCs observed with skin electrodes, but not with fiber electrodes, may be attributed to differences in the internal signal processing of the devices. The skin electrodes’ lower SNR results in stronger device internal filtering during wavelet denoising (Ahmadi and Quian Quiroga, 2013). This may enhance prominent group-specific features by smoothing the signal and reducing noise, thereby selectively revealing differences in a-wave amplitude and timing.

Our results are particularly relevant for the RETeval^®^ device. Further experiments are required to understand the underlying causes of the observed effects and to assess the transferability to other ERG devices.

Since our study focused on patients with depression, the findings cannot be extrapolated to individuals with other psychiatric disorders. Furthermore, some of the patients with MDD received antidepressant medication. Although none of the MDD patients took antidepressants for more than 14 days and it was clinically verified that remission of depression had not yet occurred, a confounding effect of antidepressant intake on the reduced amplitude and peak time of the a-wave in MDD cannot be ruled out (Moulard et al., 2022). Nevertheless, we conducted a descriptive comparison of the a-wave amplitude and peak time recorded with skin electrodes, analyzing medicated (N = 20) and unmedicated (N = 9) MDD patients separately. This subgroup analysis revealed that the mean a-wave amplitude is similarly attenuated in both medicated (vs. HCs: −14%) and unmedicated (vs. HCs: −12%) patients, while the shorting in the mean a-wave peak time was more pronounced for unmedicated (vs. HCs: −6%) than for medicated patients (vs. HCs: −2%) (Supplementary Figure S1).

Because the assumption of normal distribution was violated in some data sets, we chose non-parametric median-based approaches for hypothesis testing and effect size estimations. For comparison, and because non-parametric tests are often less powerful than parametric methods, we additionally incorporated arithmetic means and *t*-test results. Notably, for the a-wave amplitude, which exhibited a normal distribution in both groups, the two location parameters (median and mean) showed a relatively large divergence. This resulted in a more pronounced difference in group means (MDD patients vs. HCs: −13%) compared to the group medians (MDD patients vs. HCs: −5%) for the a-wave amplitude recorded with sensor strip electrodes.

In summary, the study compared sensor strip and corneal fiber electrodes in fERG examinations for HCs and patients with MDD. Despite producing smaller amplitudes compared to corneal electrodes, skin-placed sensor strip electrodes effectively detected ERG alterations in MDD patients that were not identified by fiber electrodes. Sensor strip skin, rather than corneal fiber electrodes, may be particularly suitable for RETeval^®^ fERG recordings in patients with MDD who often exhibit reduced compliance and limited attentional capacity. Prior to the implementation of skin electrodes in psychiatric samples, a replication of the study in an independent sample is necessary.

## Data Availability

The raw data supporting the conclusions of this article will be made available by the authors, without undue reservation.
