# Supplementary material for: Analysis of skin and corneal fiber electrodes for electroretinogram assessments in patients with major depressive disorder
Source: Front Neurosci. 2024 Nov 25;18:1501149. doi: 10.3389/fnins.2024.1501149 (PMC11626904; doi:10.3389/fnins.2024.1501149)
Supplement: Supplementary file 1 [file Data_Sheet_1.docx]

**Supplementary material to:**

**Analysis of skin and corneal fiber electrodes for electroretinogram assessments in patients with major depressive disorder**

**Kathrin Nickel^1*^**^†^**, Ludger Tebartz van Elst^1^**^†^**, Malina Beringer^1^, Dominique Endres^1^, Kimon Runge****^1^, Simon Maier^1^, Sebastian Küchlin^2^, Michael Bach^2^, Katharina Domschke^1,3^, Sven P. Heinrich^2#^, Evelyn B.N. Friedel^1,2,4#^**

^1^Department of Psychiatry and Psychotherapy, Medical Center – University of Freiburg, Faculty of Medicine, University of Freiburg, Freiburg, Germany

^2^Eye Center, Medical Center – University of Freiburg, Faculty of Medicine, University of Freiburg, Freiburg, Germany

^3^German Center for Mental Health (DZPG), Partner Site Berlin, Berlin, Germany

^4^Faculty of Biology, University of Freiburg, Freiburg, Germany

^†^These authors share first authorship

^#^These authors share senior authorship

**Content**

1. Supplementary Table 1: fERG parameter in MDD compared to HC.
2. Supplementary Table 2: Sensor strip vs. fiber electrodes in HC.
3. Supplementary Figure 1: Descriptive a-wave analysis in medicated and unmedicated patients with MDD compared to HC.

**Supplementary Table 1: fERG parameter in MDD compared to HC.** For patients with MDD and HC the a- and b-wave peak amplitudes and peak times, the amplitude ratio (b/a), the SNR (b-wave/noise) and the number of rejected flashes are summarized for fiber and sensor strip electrodes. Data are presented as medians, along with Wilcoxon test results and effect sizes (*r*). For comparison, means and standard deviations (SD) are provided in brackets, alongside *t*-test results and Cohen’s *d*. Departures from normality in data sets are indicated by italicized means. Confidence intervals (95%) are shown in square brackets. Significance levels in backets were adjusted according to an FDR procedure for the primary and secondary outcome variables separately. Proportional deviations of the MDD medians and means from the HC medians and means are given in % (MDD vs. HC).

| **Sensor strip** | **scale** | **MDD (N = 29)** | **HC (N = 57)** | **MDD vs. HC** | ***r/d*** | ***p–*value** |
| --- | --- | --- | --- | --- | --- | --- |
| **a-wave amplitude [µV]** | median | 5.0 [4.2, 5.4] | 5.3 [4.9, 5.9] | -5% | **0.22** | **0.020 (*)** |
|  | mean | 4.8 (1.3) [4.3, 5.3] | 5.6 (1.5) [5.2, 6.0] | -13% | **0.53** | **0.010 (*)** |
| b-wave amplitude [µV] | median | 24.1 [20.5, 27.0] | 24.7 [21.9, 26.3] | -2% | 0.11 | 0.328 (ns) |
|  | mean | 24.1 (6.3) [21.7, 26.5] | *26.1 (7.2) [24.2, 28.0]* | -8% | 0.30 | 0.186 (ns) |
| **a-wave peak time [ms]** | median | 9.9 [9.6, 10.2] | 10.2 [10.0, 10.7] | -4% | **0.26** | **0.015 (*)** |
|  | mean | *10.0 (0.7) [9.7, 10.3]* | 10.4 (0.8) [10.1, 10.6] | -3% | 0.46 | 0.043 (ns) |
| b-wave peak time [ms] | median | 27.6 [27.3, 28.1] | 28.0 [27.8, 28.2] | -1% | 0.11 | 0.293 (ns) |
|  | mean | 27.8 (0.8) [27.5, 28.1] | *28.0 (1.0) [27.8, 28.3]* | -1% | 0.22 | 0.318 (ns) |
| amplitude ratio (b/a) | median | 4.85 [4.55, 5.43] | 4.60 [4.26, 4.90] | +5% | 0.16 | 0.136 (ns) |
|  | mean | 5.15 (1.17) [4.70, 5.59] | *4.83 (1.12) [4.54, 5.13]* | +7% | 0.28 | 0.236 (ns) |
| SNR (b-wave/Noise) | median | 36.7 [24.7, 53.2] | 33.2 [28.0, 46.8] | +10% | 0.00 | 0.971 (ns) |
|  | mean | *40.4 (20.5) [32.6, 48.2]* | *46.2 (36.0) [36.6, 55.7]* | -12% | 0.20 | 0.347 (ns) |
| N flashes rejected | median | 2.5 [1.0, 3.5] | 1.0 [0.5, 2.0] | +150% | 0.27 | 0.013 (ns) |
|  | mean | *2.9 (3.2) [1.7, 4.2]* | *1.5 (1.5) [1.1, 1.9]* | +93% | 0.57 | 0.031 (ns) |
| **Fiber** | **scale** | **MDD (N = 28)** | **HC (N = 54)** | **MDD vs. HC** | ***r/d*** | ***p–*value** |
| a-wave amplitude [µV] | median | 17.9 [15.8, 19.1] | 18.6 [16.8, 20.1] | -3% | 0.09 | 0.207 (ns) |
|  | mean | 17.9 (4.0) [16.3, 19.5] | 18.6 (5.0) [17.2, 19.9] | -3% | 0.14 | 0.267 (ns) |
| b-wave amplitude [µV] | median | 75.6 [70.6, 88.5] | 77.5 [67.9, 83.7] | -2% | 0.05 | 0.649 (ns) |
|  | mean | *81.5 (24.4) [72.1, 90.9]* | 78.3 (21.5) [72.4, 84.2] | +4% | 0.14 | 0.563 (ns) |
| a-wave peak time [ms] | median | 11.2 [10.8, 11.5] | 11.4 [11.1, 11.8] | -2% | 0.13 | 0.251 (ns) |
|  | mean | 11.3 (1.0) [10.9, 11.7] | 11.4 (0.8) [11.2, 11.6] | -1% | 0.17 | 0.485 (ns) |
| b-wave peak time [ms] | median | 29.0 [28.4, 29.2] | 29.0 [28.6, 29.3] | -0% | 0.05 | 0.685 (ns) |
|  | mean | 29.0 (0.8) [28.6, 29.3] | *29.1 (1.1) [28.8, 29.4]* | -1% | 0.17 | 0.485 (ns) |
| amplitude ratio (b/a) | median | 4.86 [3.98, 5.03] | 4.18 [3.99, 4.44] | +16% | 0.14 | 0.192 (ns) |
|  | mean | 4.58 (0.84) [4.25, 4.90] | *4.36 (0.98) [4.09, 4.63]* | +5% | 0.24 | 0.296 (ns) |
| SNR (b-wave/Noise) | median | 59.0 [49.7, 75.8] | 57.6 [55.0, 70.6] | +2% | 0.00 | 0.996 (ns) |
|  | mean | *72.7 (42.6) [56.2, 89.2]* | *72.9 (41.9) [61.4, 84.3]* | -0% | 0.00 | 0.984 (ns) |
| N flashes rejected | median | 4.5 [3.0, 6.5] | 4.0 [3.0, 5.0] | +12% | 0.10 | 0.365 (ns) |
|  | mean | *5.3 (3.4) [4.0, 6.7]* | *4.8 (4.0) [3.7, 5.9]* | +11% | 0.14 | 0.548 (ns) |

Abbreviations: *d* = Cohen’s d; FDR = false discovery rate; HC = healthy controls; MDD = patients with major depressive disorder; N = number of observations; ns = not significant; *r* = robust effect size; Sensor = Sensor strip skin electrodes; SNR = signal to noise ratio (b-wave/noise); * = statistically significant.

**Supplementary Table 2.** **Sensor strip vs. fiber electrodes in HC**. For both sensor strip and fiber electrodes, peak amplitudes, and peak times of the a- and b-wave, the amplitude ratio (b/a), the SNR (b-wave/noise) and the number of rejected flashes from HC are summarized by the medians with results from the Wilcoxon tests and effect sizes (*r*). For comparison, the means and standard deviations (SD) are provided in brackets alongside *t*-test results and Cohen’s *d*. Departures from normality in data sets are indicated by italicized means. Confidence intervals (95%) are given in square brackets. Significance levels, shown in brackets, were adjusted using the FDR procedure separately for primary and secondary outcome variables. The proportional deviation of the sensor strip medians and means from the fiber electrode medians and means are given in % (Sensor vs. Fiber electrodes). Spearman’s *rho* was computed for correlation analysis.

| **Parameter** | **Scale** | **Sensor strip**  **(N=54)** | **Fiber**  **(N=54)** | **Sensor vs. Fiber** | ***r/d*** | **Sig. level** | **correlation** | |  |
| --- | --- | --- | --- | --- | --- | --- | --- | --- | --- |
|  |  |  |  |  |  |  | ***rho*** | ***p–*value** |  |
| a-wave amplitude [µV] | median | 5.4 [5.0, 5.9] | 18.6 [16.8, 20.1] | -71% | 0.87 | *** | 0.33 | 0.015 (*) | |
|  | mean | 5.6 (1.5) [5.2, 6.0] | 18.6 (5.0) [17.2, 19.9] | -70% | 2.78 | *** |  |  | |
| b-wave amplitude [µV] | median | 24.8 [22.7, 26.3] | 77.5 [67.9, 83.7] | -68% | 0.87 | *** | 0.50 | <0.001 (*) | |
|  | mean | *26.3 (7.3) [24.3, 28.3]* | 78.3 (21.5) [72.4, 84.2] | -66% | 2.82 | *** |  |  | |
| a-wave peak time [ms] | median | 10.3 [10.1, 10.7] | 11.4 [11.1, 11.8] | -10% | 0.87 | *** | 0.79 | <0.001 (*) | |
|  | mean | 10.4 (0.8) [10.2, 10.6] | 11.4 (0.8) [11.2, 11.6] | -9% | 1.93 | *** |  |  | |
| b-wave peak time [ms] | median | 28.0 [27.9, 28.2] | 29.0 [28.6, 29.3] | -3% | 0.87 | *** | 0.74 | <0.001 (*) | |
|  | mean | *28.1 (1.0) [27.8, 28.4]* | *29.1 (1.1) [28.8, 29.4]* | -4% | 1.57 | *** |  |  | |
| amplitude ratio (b/a) | median | 4.56 [4.26, 4.87] | 4.18 [3.99, 4.44] | +9% | 0.51 | *** | 0.69 | <0.001 (*) | |
|  | mean | *4.82 (1.13) [4.51, 5.13]* | *4.36 (0.98) [4.09, 4.63]* | +11% | 0.52 | *** |  |  | |
| SNR (b-wave/Noise) | median | 33.8 [29.3, 48.2] | 57.6 [55.0, 70.6] | -41% | 0.51 | *** | 0.34 | 0.011 (*) | |
|  | mean | *47.5 (36.5) [37.6, 57.5]* | *72.9 (41.9) [61.4, 84.3]* | -35% | 0.49 | *** |  |  | |
| N flashes rejected | median | 1.0 [0.5, 1.5] | 4.0 [3.0, 5.0] | -75% | 0.74 | *** | 0.35 | 0.011 (*) | |
|  | mean | *1.5 (1.5) [1.1, 1.9]* | *4.8 (4.0) [3.7, 5.9]* | -69% | 0.87 | *** |  |  | |

Abbreviations: *d* = Cohen’s *d*; FDR = false discovery rate; N = number of healthy controls (HC); ns = not significant; *r* = robust effect size; *rho* = Spearman’s correlation coefficient; Sensor = Sensor strip electrode; SNR = signal to noise ratio (b-wave/noise); * = statistically significant; *** = *p*–value <0.001.

**
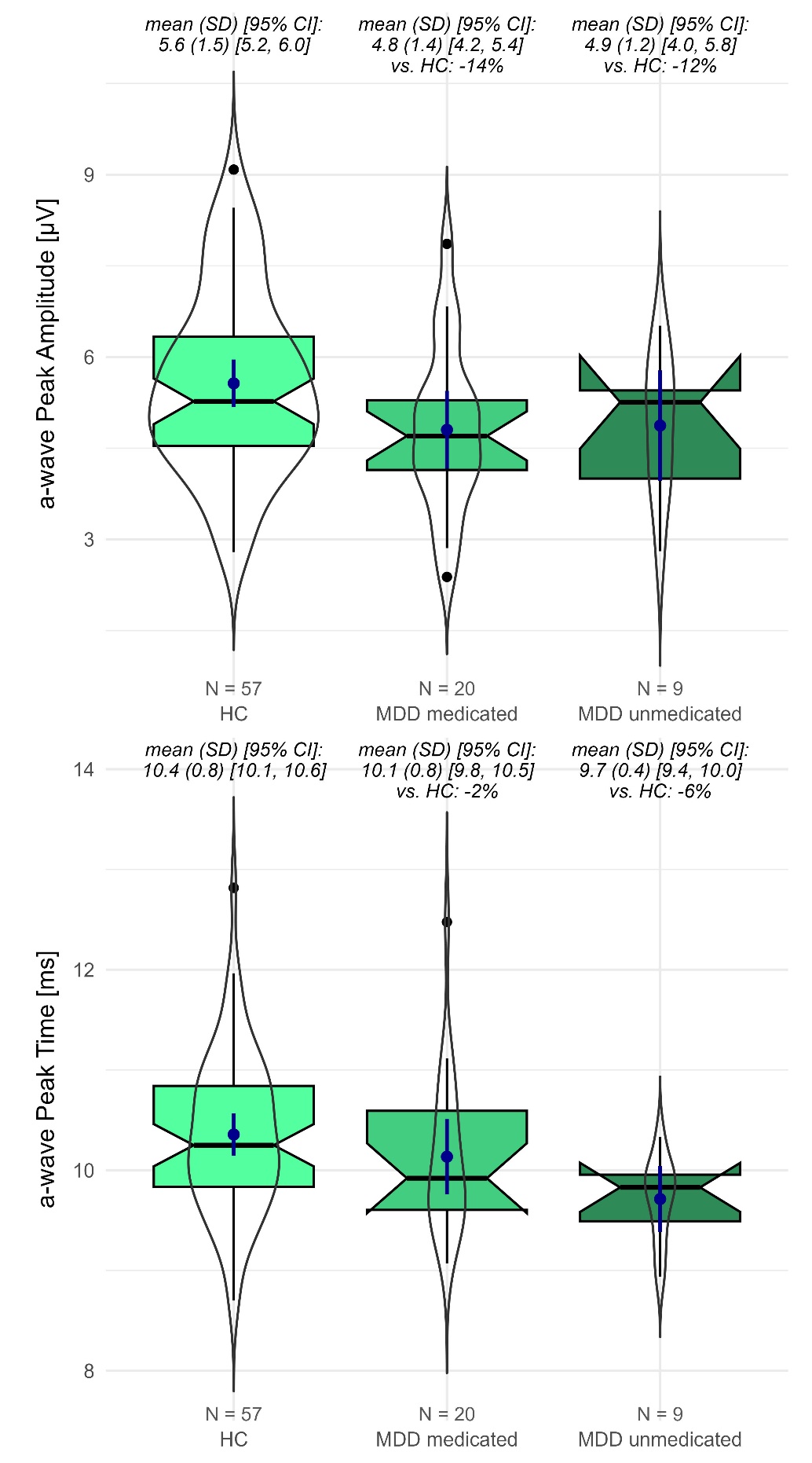
**

**Supplementary Figure 1: Descriptive a-wave analysis in medicated and unmedicated patients with MDD compared to HC.** A-wave amplitude (upper panel) and peak time (lower panel) for medicated and unmedicated patients with MDD in descriptive comparison to HC. The means, standard deviations (SD) and 95% confidence intervals are annotated along with the relative deviation in % of the patient’s means from the HC means (vs. HC). Both medicated and unmedicated patients with MDD showed attenuations in the a-wave amplitude recorded with sensor strip electrodes. The reduction in the peak time of the a-wave recorded with the skin electrodes is more pronounced in unmedicated compared to medicated patients. Abbreviations: HC = healthy controls; N = number of observations; MDD = patients with major depressive disorder; SD = standard deviation.
